# What are we really measuring? The semantic confound in psychological scales and the case for theory-driven measurement

**DOI:** 10.3389/fpsyg.2026.1870659

**Published:** 2026-08-11

**Authors:** Xiangyu Liu

**Affiliations:** Yuankong Technology Co., Ltd., Beijing, China

**Keywords:** construct validity, holographic coding framework, psychological measurement, semantic confound, theory-driven measurement

## Abstract

The replication crisis in psychology is now widely recognized as a crisis of measurement itself, not merely of questionable research practices. Recent critical work has revealed that many psychological scales may be measuring semantic associations rather than the psychological phenomena they purport to assess. This Hypothesis and Theory article makes three contributions. First, it synthesizes diverse lines of critique—including Uher’s distinction between statistical modeling and measurement, and the finding that computational language models, trained solely on word co-occurrence patterns, can predict questionnaire responses with surprising accuracy—without any access to human participants’ psychological states. These critiques converge on a common diagnosis: the “semantic confound,” where scales capture linguistic familiarity rather than latent psychological traits. Second, it argues that the root cause of this measurement crisis is not statistical but theoretical: the absence of explicit, falsifiable theories about the target phenomena themselves. This theory vacuum has, in practice, allowed scale development to default to an inductive, data-driven approach that conflates statistical structure with psychological structure, despite the field’s long-standing theoretical commitment to construct validity (Cronbach and Meehl, 1955). Third, it outlines what theory-driven measurement would require: axiomatic assumptions, phenomenologically distinct state definitions, formal causal structures, and a mapping function from observable behaviors to latent states. We demonstrate that these conditions are neither arbitrary nor utopian by examining four research traditions—psychophysics, structured diagnostic interviews, item response theory, and cognitive diagnostic models—that each partially satisfy one or more of them. The Holographic Coding Framework (HCF), recently proposed as a theory-driven approach to consciousness measurement, is briefly introduced as an illustrative case of this alternative paradigm. We conclude by calling for a fundamental shift in quantitative psychology: from measurement models that fit data to theories that drive measurement. Standardizing poor measurement entrenches the validity crisis; theorization must precede standardization.

## 1 Introduction

The replication crisis in psychology has, for over a decade, prompted intense methodological self-scrutiny. Researchers have identified questionable research practices (John et al., 2012), advocated for pre-registration (Nosek et al., 2018), and called for larger sample sizes and higher power (Button et al., 2013). These reforms, while valuable, address the symptoms rather than the root cause. As the editors of a recent special issue in this journal have argued, “questionable research practices are just surface-level symptoms of much more profound issues” rooted in inadequate ontological, epistemological, and methodological foundations (Uher et al., 2025b). In a companion article in the same special issue, Uher et al. (2025a) developed a taxonomy of “Questionable Research Fundamentals” (QRFs) to systematically catalog the deeper, ontological and epistemological roots of the crisis.

Among these foundational issues, measurement is perhaps the most neglected. Psychologists routinely administer scales, compute Cronbach’s alpha, and report factor loadings, yet rarely ask a more fundamental question: what, exactly, are these scales measuring? The assumption has been that a scale labeled “emotional acceptance” measures emotional acceptance, and that internal consistency and factor structure suffice to establish this. Recent work challenges this assumption at its core.

Karvelis and Diaconescu (2025), also in this journal, demonstrated that measurement unreliability—a problem long acknowledged but seldom quantified—can severely attenuate group differences, undermining our ability to detect effects even when they exist. This “reliability paradox” is only one facet of a deeper problem. Hentati Isacsson et al. (2026) documented that “the dominant measurement practices in psychology often lack rigor,” noting that researchers “often ignore issues regarding measurement” and “seldom motivate the validity of instruments.” These critiques converge on an uncomfortable conclusion: the problem is not that our measurements are imprecise, but that they may be measuring something entirely different from what we think they are measuring.

This conceptual analysis advances this critical conversation by synthesizing multiple lines of evidence into a single, coherent argument. We first examine the “semantic confound”—the finding that many psychological scales capture linguistic familiarity rather than psychological states. We then argue that the root cause is a theory vacuum: psychology has developed increasingly sophisticated statistical tools for scale construction while neglecting the theoretical foundations that give measurements their meaning. Finally, we outline what theory-driven measurement would require and briefly introduce one such attempt—the Holographic Coding Framework (HCF; under review)—as an illustration of what theory-driven measurement might look like in practice. The framework is not presented as the solution, but as a concrete example that helps make the abstract principles of theory-driven measurement more tangible. The principles themselves are independent of any single framework’s empirical success.

This paper does not claim that all existing scales are invalid. It does not claim that the HCF has solved the measurement crisis. It does not claim that statistical models (IRT, Rasch) are useless. It claims that measurement validity requires theoretical grounding, and that this grounding has been neglected.

## 2 The semantic confound: what scales actually measure

### 2.1 Statistics is not measurement

The distinction between statistical modeling and measurement, while fundamental to the philosophy of science, is rarely observed in psychological practice. Uher (2025), in the special issue that frames this discussion, articulates this distinction with precision: statistical modeling concerns relationships among data points—correlations, factor loadings, regression coefficients—regardless of what those data represent. Measurement, by contrast, requires establishing “traceable empirical relations” between the property being measured and the numbers assigned to it.

A scale can exhibit excellent psychometric properties—high internal consistency, clear factor structure, strong test–retest reliability—and still fail as a measurement instrument if these numerical properties are not demonstrably linked to the target phenomenon itself. The thermometer does not work because mercury expands and contracts; it works because this expansion and contraction has been traced to the thermal energy of the substance being measured. Psychology has, by and large, insufficiently formalized this tracing step. Traditional scale development is often guided by implicit theoretical assumptions rather than explicit, falsifiable theories about the target phenomenon. The issue is not an absence of theory, but a lack of formal, testable theory prior to item generation. We have sophisticated statistical models of scale responses, but we lack correspondingly sophisticated models of how those responses relate to the psychological states they are supposed to indicate.

### 2.2 Language models can predict questionnaire responses without human participants

This is not a hypothetical problem. More than a decade ago, Arnulf et al. (2014) provided the first empirical demonstration that the semantic properties of questionnaire items could predict response structures without any reference to the psychological states of respondents. Analyzing large-scale survey data on organizational behavior, they showed that purely semantic algorithms, trained on word co-occurrence patterns, could reproduce the observed response distributions with surprising accuracy—challenging the assumption that such distributions reflect latent psychological traits rather than linguistic regularities.

More recently, Ravenda et al. (2025) substantiated and extended this finding using large language models (LLMs). Their analyses confirmed that questionnaire structures are substantially shaped by item wording and linguistic associations, often to a degree that rivals or exceeds the influence of the latent psychological constructs the scales are intended to measure. The authors concluded with a call for “a re-examination of what and how we truly measure in psychology” (Ravenda et al., 2025)—a conclusion that aligns closely with Uher’s (2025) diagnosis that statistical models of verbal behavior have been mistaken for measurement of psychological phenomena.

This line of evidence has profound implications for psychological measurement. If a language model, devoid of emotions, consciousness, or any psychological life whatsoever, can produce the same response patterns we interpret as indicators of psychological states, then what exactly are our scales measuring? The parsimonious interpretation is that they measure—at least in part—the semantic associations among words, not the psychological phenomena those words are intended to denote.

This finding alone does not imply that a scale is invalid. Questionnaire items are necessarily linguistic artifacts, and their semantic properties are part of their communicative function (Tourangeau et al., 2000). The concern arises when a scale offers no additional, empirically supported predictions beyond what semantic associations alone can explain. The semantic confound is not that items have semantic structure—it is that this structure is mistaken for psychological structure when the scale lacks a theoretical framework that generates predictions beyond linguistic regularities.

This is not merely a methodological curiosity. It strikes at the heart of what we take psychological measurement to be. When a participant endorses the item “I am able to accept my emotions,” their response reflects at least two things: their reading comprehension of the sentence, and whatever psychological state the sentence is intended to probe. The language model reproduces the first without the second, yet produces indistinguishable aggregate results.

### 2.3 The AAQ-II as a case in point

Consider the Acceptance and Action Questionnaire–II (AAQ-II; Bond et al., 2011), one of the most widely used measures of psychological inflexibility and experiential avoidance—a construct closely related to emotional acceptance. The AAQ-II asks participants to rate statements such as “I’m afraid of my feelings” and “I worry about not being able to control my worries and feelings.” High scores are interpreted as indicating high experiential avoidance.

But what is actually being measured? The item “I’m afraid of my feelings” measures, at minimum, the participant’s willingness to endorse the statement “I’m afraid of my feelings.” The inference from this endorsement to the psychological state of experiential avoidance requires a chain of auxiliary assumptions: that participants have introspective access to their emotional avoidance, that they are willing to report it accurately, that the language of the item maps onto their experience in a consistent way, and that the latent construct of “experiential avoidance” is causally responsible for the observed response. None of these assumptions is tested by calculating Cronbach’s alpha.

This is not merely a conceptual possibility. Research has documented that AAQ-II scores show high correlations with other self-report measures of emotional distress but modest correlations with behavioral measures of experiential avoidance(Wolgast, 2014; Tyndall et al., 2019), suggesting that what the scale measures may not be what its label claims.

The distinction is not merely academic. The same AAQ-II score could reflect genuinely high experiential avoidance, a self-critical response style, cultural norms about emotional expression, or simply the participant’s semantic understanding that “being afraid of feelings” is something people report when they are distressed. Without a theory that specifies how experiential avoidance manifests in observable behavior—beyond questionnaire responses—we cannot distinguish among these possibilities.

### 2.4 The semantic roots of the jingle-jangle fallacy

The persistence of the jingle-jangle fallacy reflects multiple contributing factors, including sociocultural conventions (different research traditions using different terms for similar constructs), disciplinary fragmentation (lack of communication across subfields), and—central to our argument—semantic confound (item wording driving factor structures independently of psychological content). This manuscript focuses on the latter, which has received less attention in the literature.

The Jingle-Jangle fallacy—where different constructs share the same name (jingle) or the same construct goes by different names (jangle)—is a well-known problem in psychological measurement (Kelley, 1927; Block, 1995), while different constructs often share overlapping item content due to shared linguistic conventions rather than shared psychological structure. Its persistence, we suggest, reflects a deeper semantic confusion. Traditional scale development cannot resolve the jingle-jangle problem because it relies on the very semantic associations that create it.

When scales are developed through factor analysis of item pools, the resulting factors reflect patterns of linguistic co-occurrence as much as patterns of psychological co-occurrence. Two sets of items may load on different factors not because they tap different psychological processes, but because they employ different vocabularies. Conversely, items with similar wording may load on the same factor regardless of their psychological content. The result is a taxonomy of psychological constructs that is, in part, a taxonomy of linguistic habits.

### 2.5 Defining the semantic confound: three variants

The semantic confound refers to the phenomenon where scale scores reflect linguistic regularities rather than the psychological constructs they purport to measure. The semantic confound can manifest in several ways, which we organize into three distinct variants for analytical clarity. We distinguish three variants:

*Lexical confound*: Different constructs share similar wording (e.g., “emotional acceptance” and “cognitive reappraisal” may use overlapping terms), leading to spurious correlations.

*Pragmatic confound*: The same wording maps to different psychological states across contexts (e.g., “I accept my feelings” may mean different things to a clinically depressed vs. a non-clinical individual).

*Referential confound*: Scale scores reflect familiarity with linguistic conventions (e.g., how words co-occur in natural language) rather than the target psychological state.

Operational criterion: If a language model trained solely on word co-occurrence patterns…can reproduce a substantial portion of the response variance in a scale (for heuristic purposes, we adopt ≥80% as a provisional threshold; cf. Landauer and Dumais, 1997, on semantic space conventions), this is taken as evidence that the scale may be susceptible to semantic confound. This threshold is proposed as a heuristic benchmark for identifying potential cases worthy of further investigation—not as a definitive test of invalidity, and not as a psychometrically derived cutoff. It is falsifiable and open to refinement based on future empirical work.

## 3 Why this happened: the theory vacuum

### 3.1 Psychology’s anti-theory tendency

The semantic confound in psychological measurement did not arise by accident. It is a predictable consequence of psychology’s long-standing ambivalence toward theory. Since the cognitive revolution, psychology has accumulated empirical findings at an accelerating rate, but its theoretical foundations have not kept pace. Major theoretical frameworks—behaviorism, psychoanalysis, cognitivism—have been largely replaced not by better theories but by statistical models that describe data without explaining the phenomena that generate them. This is not to deny the field’s rich tradition of theoretical work on validity itself. Construct validity (Cronbach and Meehl, 1955; Messick, 1989) and subsequent argument-based approaches (Kane, 2006, 2013; see also Borsboom, 2008, on the conceptual status of latent variables) have provided essential frameworks for evaluating whether a test measures what it purports to measure. However, a gap remains between these theoretical principles and their implementation in routine scale development—a gap where statistical convenience often fills the void left by insufficiently formalized theory.

This anti-theory tendency has been noted by multiple observers. Meehl (1978) warned decades ago that “theories in ‘soft’ areas of psychology” were being “refuted” not by strong tests but by the accumulation of null results from weak ones. Discussions of this crisis have been particularly prominent in European psychology, where scholars have examined its historical roots and epistemological dimensions (Gigerenzer, 2025a,b). The question of whether psychology’s theoretical fragmentation reflects a deeper crisis of scientific reasoning has also been explored from a metatheoretical perspective (Slunecko and Wieser, 2021). More broadly, the need to move beyond descriptive models toward formal, testable theories has been identified as a priority for the field (Borsboom et al., 2024). More recently, Oberauer and Lewandowsky (2019) argued that psychology suffers from a “theory crisis” distinct from and deeper than its replication crisis. The editorial framing of the special issue in this journal (Uher et al., 2025b) places this crisis at the level of ontology and epistemology, not merely methodology.

### 3.2 Measurement without theory

In practice, despite the field’s established theoretical frameworks for validation (Cronbach and Meehl, 1955; Messick, 1989; Kane, 2013), scale development has often defaulted to an inductive approach: generate a pool of items, administer them to a sample, extract factors, and label the result. This is not because validity theory does not exist, but because the theoretical grounding of constructs is frequently undertheorized prior to item generation, leaving statistical convenience to fill the gap. This approach, while practical, conflates statistical structure with psychological structure.

The result is a measurement literature in which constructs are defined by their measures rather than the reverse. “Emotional acceptance” is what the AAQ-II measures; the AAQ-II measures emotional acceptance. This circularity immunizes measurement from theoretical critique—there is no independent specification of the construct against which the measure can be evaluated—but it also leaves us unable to say what exactly we are measuring or why it matters.

### 3.3 The Michell critique: psychometrics as pathological science

The most radical version of this critique comes from Joel Michell, who argued across several decades that psychometrics is a “pathological science” in precisely the sense defined by Langmuir (Michell, 1997, 2000, 2008). The core of Michell’s argument is that psychometricians have systematically avoided the fundamental question of whether psychological attributes are quantitative—that is, whether they possess a structure that warrants numerical measurement at all.

Michell’s critique has been largely ignored rather than refuted. For our purposes, it highlights a crucial point: psychological measurement, as currently practiced, rests on assumptions about the nature of its objects that have never been adequately defended. We assume that emotional acceptance, anxiety, and depression are continuous quantities amenable to interval-level measurement, not because we have established this through theoretical analysis and empirical demonstration, but because this assumption allows us to use the statistical tools at our disposal.

### 3.4 The language model experiment as a “faking” test

Returning to the language model findings of Arnulf et al. (2014) and Ravenda et al. (2025) discussed in Section II, we can now see their full significance. The language model is, in effect, performing a “faking” test: it demonstrates that response patterns we attribute to psychological causes can be reproduced by a mechanism that operates on purely linguistic principles. A good measurement instrument should fail this test—its responses should not be reproducible without the psychological phenomenon it purports to measure.

The fact that many scales pass the language model test (i.e., their responses can be reproduced without participants) does not prove that these scales are worthless. But it does shift the burden of proof. It is no longer sufficient to demonstrate that a scale has good psychometric properties; we must also demonstrate that its responses are not merely reflecting linguistic regularities. This requires a theory of the target phenomenon that makes predictions beyond what semantic associations alone can explain.

## 4 What theory-driven measurement would look like

The preceding sections diagnosed the semantic confound as a fundamental threat to validity in psychological measurement and proposed four necessary conditions for theory-driven measurement. This section introduces the Holographic Coding Framework (HCF)—a theory that satisfies these four conditions and offers a coherent alternative to inductive scale development. The HCF is not merely an illustration; it is the alternative view called for in the critique.

### 4.1 Necessary conditions for theory-driven measurement

If the semantic confound arises from measuring without theory, the solution is measurement driven by theory. Drawing on the preceding analysis, achieving such grounding requires meeting four interrelated conditions. We propose four necessary conditions for theory-driven measurement in psychology:

*Axiomatic foundations*: The theory must begin with explicit, stated assumptions about the nature of the target phenomenon—what it is, how it operates, and what its essential properties are. These axioms are not themselves tested; they define the framework within which testing occurs.

*Phenomenologically distinct state definitions*: The theory must specify a set of distinct states that the target system can occupy, with clear criteria for distinguishing among them. These states must be defined independently of the measurement instrument.

*Formal causal structure*: The theory must specify how states relate to one another causally, including direction, strength, and conditions under which relationships hold. This structure generates testable predictions.

*Traceable mapping function*: The theory must specify how observable behaviors—including but not limited to verbal reports—map onto the latent states. This mapping constitutes the “traceable empirical relation” that Uher (2025) identifies as the hallmark of genuine measurement.

These conditions are demanding, and it is unlikely that any existing psychological measure fully satisfies them. But they represent a direction, not a threshold. The question is not whether our current measures are perfect but whether we are moving toward measurement that is increasingly grounded in theory rather than in statistical convenience.

Together, these four conditions specify what Uher (2025) calls “traceable empirical relations”—a verifiable link between the theoretical construct and the observed data.

Each of these conditions responds directly to a specific deficiency identified in Section II. Axiomatic foundations (Condition 1) address the theory vacuum described in Section III—without explicit theoretical commitments, measurement defaults to statistical modeling. Phenomenologically distinct state definitions (Condition 2) address the semantic confound identified in Section II: if a construct is defined only by its scale items, we cannot distinguish measurement of the construct from measurement of the linguistic associations among those items. Formal causal structure (Condition 3) addresses the Jingle-Jangle fallacy—causal relations among constructs are independent of their names, providing an empirical basis for distinguishing genuinely different constructs from semantically related ones. Traceable mapping (Condition 4) directly addresses Uher’s (2025) requirement for “traceable empirical relations”—the hallmark of genuine measurement that distinguishes it from mere statistical modeling (see Table 1).

**Table 1:** How the four necessary conditions address the semantic confound.

Condition	Problem addressed	How it prevents semantic confound
Axiomatic foundations	Construct defined post-hoc by items	Forces explicit assumptions about the target phenomenon before measurement
Phenomenologically distinct states	Construct conflated with linguistic labels	Requires state definitions independent of measurement instruments
Formal causal structure	Spurious correlations between semantically related constructs	Specifies directional, testable relationships that cannot be reduced to word co-occurrence
Traceable mapping function	No verifiable link between behavior and latent state	Requires observable behaviors that are demonstrably linked to the theoretical construct

### 4.2 Precursors and partial satisfactions

The four conditions we have proposed are demanding, but they are not without precedent. Several established research traditions have partially satisfied one or more of them, though none has fully integrated all four into a unified measurement framework. Examining these precursors serves two purposes: it grounds our proposal in the history of psychological science, and it clarifies what is genuinely new about the theory-driven measurement paradigm we advocate.

#### 4.2.1 Psychophysics and the traceable mapping of sensation

The methods of psychophysics—signal detection theory, magnitude estimation, and cross-modal matching—achieved something that most contemporary psychological measurement has not: they established traceable, quantitative relations between physical stimulus properties and subjective experience. Stevens’ power law (*ψ* = *k*·*φ^n^*), for instance, formalized the mapping between stimulus intensity (*φ*) and perceived sensation magnitude (*ψ*). This mapping is traceable in precisely the sense Uher (2025) described: the correspondence between the physical property and the psychological one is empirically demonstrated, not merely assumed. Psychophysics thus satisfies a core requirement of theory-driven measurement. Its limitation, for our purposes, is that it has been largely confined to simple perceptual dimensions—brightness, loudness, heaviness—and has not been extended to the complex, multi-dimensional constructs (emotional acceptance, cognitive congruence, goal-directed efficacy) that constitute the subject matter of clinical and personality psychology (see Table 2).

**Table 2:** How the HCF satisfies the four necessary conditions.

Condition	How HCF satisfies it
Condition 1: axiomatic foundations	HCF has four explicit axioms: intrinsic worth (*K* ≡ 1), dual-layer architecture (consciousness and carrier subsystems), embodiment (consciousness manifests through and reshapes its carrier), and asymmetric influence (top-down influence is stronger than bottom-up for intentional change).
Condition 2: phenomenologically distinct states	HCF defines four *α*-factors (*α*_1_: metacognitive clarity, *α*_2_: emotional acceptance, α_3_: cognitive-behavioral congruence, α_4_: goal-directed efficacy) and four carrier factors (a: material-structural body, b: liquid-energetic body, c: gaseous-emotional body, d: electromagnetic-informational body). These are defined independently of any measurement instrument.
Condition 3: formal causal structure	HCF specifies a directed causal chain *α*_1_ → *α*_2_ → *α*_3_ → *α*_4_, which generates testable predictions (e.g., *H*_trend = [*α*_1_]^2^).
Condition 4: traceable mapping function	HCF maps observable behavioral responses (20 items) to energy codes, then to *α*-factors and carrier factors, and finally to *H* = *R*_carrier × *R*_consciousness. This mapping is empirically verifiable.

#### 4.2.2 Structured diagnostic interviews and phenomenological state definitions

In clinical psychology, structured diagnostic interviews such as the SCID represent an instructive precedent. These instruments do not ask patients to rate “how depressed” they feel on a numerical scale and then apply a cutoff. Instead, they specify in advance a set of phenomenological criteria—low mood, anhedonia, sleep disturbance, fatigue, worthlessness, diminished concentration, and suicidal ideation—and operationalize each criterion through targeted questions. A depressive episode is defined by the presence of a specific configuration of these indicators, persisting for a specified duration and causing functional impairment. The state definition is logically and temporally prior to its measurement. The limitation is that diagnostic criteria are arrived at by expert consensus rather than derived from axiomatic theory, and the resulting states are categorical (present/absent) rather than continuous, making them poorly suited to tracking gradual change.

#### 4.2.3 Item response theory and the formalization of latent traits

Item Response Theory (IRT) and Rasch Measurement Theory (RMT) represent the most sophisticated formal structures currently available in psychological measurement. They model the relationship between a latent trait (*θ*) and observed item responses through mathematical functions that account for item difficulty, discrimination, and guessing. In this respect, they go further than any other widely used method toward satisfying the “traceable mapping” condition. However, IRT and RMT take the latent trait as given; they do not derive it from independent theoretical commitments. The trait is whatever the model estimates it to be, and its psychological meaning is supplied *post hoc* through the labeling of items. This is theory-as-interpretation, not theory-as-foundation. Moreover, because IRT models treat the latent trait as a continuous dimension—analogous to a physical quantity—they inherit Michell’s (1997, 2000, 2008) challenge: whether psychological attributes possess the quantitative structure that such models presuppose is precisely what has not been established.

It is important to acknowledge the measurement assumptions underlying IRT and RMT, including uni-dimensionality (all items measure a single latent trait), local independence (item responses are independent conditional on the trait), and monotonicity (the probability of endorsing an item increases with the trait level). These assumptions are rarely tested directly in psychological applications, and violations can lead to biased parameter estimates (Maraun, 1996).

More fundamentally, as Borgstede and Eggert (2023) argue, IRT and RMT treat the latent trait as a mathematical convenience rather than a theoretically specified entity. The trait is whatever the model estimates it to be, and its psychological meaning is supplied post-hoc through item labeling. This is “theory-as-interpretation” rather than “theory-as-foundation.” Our proposal for theory-driven measurement builds on this critique by requiring that the latent construct be specified independently of the measurement instrument.

The conceptual status of latent variables in IRT and RMT is itself a matter of ongoing debate. Borsboom (2008) has argued that psychological variables should be considered latent until proven observed, and that the inference from observed responses to latent constructs is inherently epistemic rather than ontological. The present proposal does not challenge this view directly, but argues that the theoretical specification of latent constructs can be made more formal and testable prior to item generation—a step that IRT and RMT, as statistical frameworks, do not themselves require.

#### 4.2.4 Cognitive diagnostic models and discrete state definitions

In educational measurement, cognitive diagnostic models (CDMs) offer a closer approximation to theory-driven measurement. CDMs first define a set of discrete cognitive attributes—for example, “carrying in addition” or “finding a common denominator”—and then infer each student’s attribute mastery profile from their pattern of correct and incorrect responses. The attributes are defined before the items are written, and the mapping from observed responses to latent states is explicit and falsifiable. This logic parallels what we advocate for consciousness measurement: define the states first, then design the measurement. CDMs remain, however, largely confined to educational and cognitive assessment, and their attribute definitions are derived from expert task analysis rather than from axiomatic foundations.

Taken together, these precursors suggest that the conditions we have identified are neither arbitrary nor utopian. They represent natural, convergent developments in the history of psychological science—each a partial step toward a measurement paradigm that begins with what we want to measure rather than with the instruments we happen to have. What remains is the integration of these partial advances into a unified framework: axiomatic foundations from which phenomenological state definitions are derived, a formal causal structure linking those states, and a traceable mapping from observable behavior to the states thus defined. The Holographic Coding Framework, introduced below, represents one attempt at such integration.

### 4.3 Traditional scale-based measurement vs. theory-driven measurement

Before presenting the comparison, it is worth clarifying what each paradigm assumes about the nature of measurement.

Table 3 provides a schematic comparison between traditional scale-based measurement and theory-driven measurement.

**Table 3:** Traditional scale-based measurement vs. theory-driven measurement.

Dimension	Traditional scale-based measurement	Theory-driven measurement
Starting point	Item pool, factor analysis	Axiomatic assumptions about target phenomenon
Construct definition	What the scale measures	What the theory specifies
Validity evidence	Internal consistency, factor structure, correlations with other scales	Correspondence between theoretical predictions and observed behavioral patterns
Relationship to language	Inseparable from item wording	Verbal responses are one behavioral indicator among many
Generalization	Limited to populations and contexts where factor structure holds	Generalizes to any context where the theory’s predictions can be tested

### 4.4 The HCF as an illustrative case

The Holographic Coding Framework (HCF; under review) is a recently proposed framework that attempts to implement theory-driven measurement. Whether it succeeds is an empirical question; our interest here is in its structural features as an illustration of the alternative paradigm. The HCF begins with four explicit axioms: intrinsic worth (every living system possesses complete intrinsic worth), dual-layer architecture (consciousness and carrier subsystems interact), embodiment (consciousness manifests through and reshapes its carrier), and asymmetric influence (top-down influence is stronger than bottom-up).

From these axioms, the framework derives a set of core equations. Consciousness brightness (*R*_consciousness) is decomposed into four *α*-factors—meta-cognitive clarity (*α*_1_), emotional acceptance (*α*_2_), cognitive-behavioral congruence (*α*_3_), and goal-directed efficacy (*α*_4_)—linked by a directed causal chain (*α*_1_ → *α*_2_ → *α*_3_ → *α*_4_). Carrier integration (*R*_carrier) is decomposed into four carrier factors (a–d). The framework generates a falsifiable growth prediction: *H*_trend = [*α*_1_]^2^.

Crucially, the HCF does not measure α-factors by asking participants whether they possess them. Instead, it derives factor values from behavioral choices through a coding system that maps observable response patterns onto theoretically defined system states. This mapping relation—not the statistical properties of the resulting scores—is the measurement model. The framework’s theoretical derivation and initial empirical exploration are reported elsewhere; our purpose here is not to evaluate the HCF but to note that it exemplifies a different approach to measurement, one that begins with theory rather than data. While the HCF provides one concrete illustration of theory-driven measurement, the principles outlined in Sections 4.1 and 4.2 are general—they do not depend on any single framework’s success, and other instantiations are both possible and welcome (see Table 4).

**Table 4:** Operational definitions of α-factors and carrier factors.

Factor	Definition	Corresponding paper concept	Source
*α*_1_	Metacognitive clarity—system’s ability to monitor and recognize its own state	Recognition/Reverence	Synthesized from 20-item response patterns
*α*_2_	Homeostatic maintenance—system’s ability to maintain core function under interference	Acceptance/Gratitude	Synthesized from 20-item response patterns
*α*_3_	System internal consistency index (*I*)—degree of synergy among system modules	Self-consistency/Harmony	*I* = 1/(1 + Σ conflict intensity)
*α*_4_	Goal achievement efficacy—efficiency in executing preset tasks	Action/Creation	Synthesized from 20-item response patterns
a-carrier	Solid-state carrier: basic physical structure, metabolic and inflammatory status	Material-structural body	Physiological indicators/self-report
b-carrier	Liquid-state carrier: autonomic nervous system, blood circulation, energy metabolism	Liquid-energetic body	Physiological indicators/self-report
c-carrier	Gas-state carrier: respiration, emotion, physiological signals of the sensing body	Gaseous-emotional body	Physiological indicators/self-report
d-carrier	Electromagnetic/information carrier: neural signal integration, brain network coordination	Electromagnetic-informational body	Physiological indicators/self-report

The Holographic Coding Framework (HCF) is built on four axioms (see Author, under review, for full derivation):

Intrinsic worth (*K* ≡ 1): Every living system possesses complete intrinsic worth as a theoretical baseline.

Dual-layer architecture: Living systems consist of interacting consciousness and carrier subsystems.

Embodiment: Consciousness manifests through and reshapes its carrier.

Asymmetric influence: Top-down (consciousness → carrier) influence is stronger than bottom-up for intentional change.

From these axioms, the framework derives core equations:

H=R_carrier×R_consciousness

$$R\_\text{carrier}={\left(a\cdot b\cdot c\cdot d\right)}^{\left\{1/4\right\}}$$

$$R\_\text{consciousness}={\left(\alpha_{1}\cdot \alpha_{2}\cdot \alpha_{3}\cdot \alpha_{4}\right)}^{\left\{1/4\right\}}$$

$$H\_\text{trend}={\left[\alpha_{1}\right]}^{2}$$

All *α*-factors and carrier factors are normalized to the [0, 1] interval, so *H* and *H*_trend also range from 0 to 1. *H*_trend represents the system‘s latent growth potential: a small increase in *α*_1_ yields a quadratic increase in *H*_trend, making *α*_1_ the most efficient target for intervention. This prediction is absent from traditional descriptive scales.

Empirical support for these predictions has been obtained in large-scale population studies (NHANES, *N* = 32,847; Author, under review). Ongoing precision validation is being conducted to further test the framework’s predictions.

**Figure 1:**
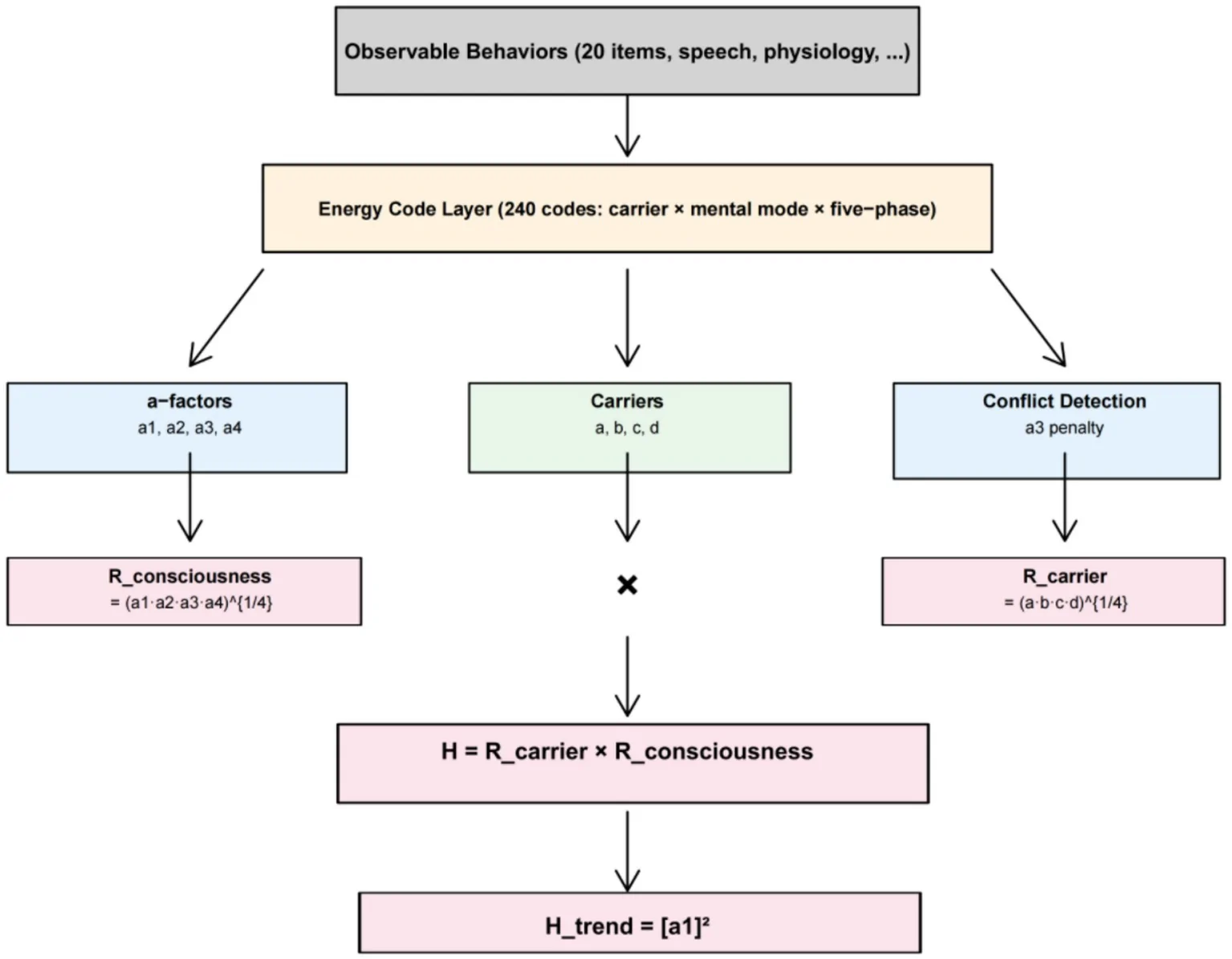
Theoretical architecture of the holographic coding framework (HCF). Observable behaviors (20 items, speech, physiology, etc.) are mapped to an energy code layer (240 codes), which then feeds into three parallel subsystems: *α*-factors (*α*_1_, *α*_2_, *α*_3_, *α*_4_), carriers (a, b, c, d), and conflict detection (which penalizes *α*_3_). These are aggregated into *R*_consciousness and *R*_carrier via geometric means, then combined multiplicatively to yield H (life fluency). Finally, *H*_trend = [*α*_1_]^2^ represents the system’s latent growth potential. All *α*-factors and carrier factors are normalized to the [0, 1] interval.

Figure 1 provides a visual summary of the HCF theoretical architecture, illustrating the mapping from observable behaviors to *H* and *H*_trend.

BOX 1 **Hypothetical calculation example for *H* and *H*_trend**

Step 1: User completes 20 items → response pattern (e.g., Q5 = 5, Q6 = 1, Q13 = 3, Q18 = 4, etc.)

Step 2: Map responses to energy codes via pre-defined mapping table.

Step 3: Compute user weights for each activated code.

Step 4: Calculate *f* (*α*_1_), *f* (*α*_2_), *f* (*α*_3_), *f* (*α*_4_) via sigmoid transformation.

Step 5: *R*_consciousness = (*α*_1_·*α*_2_·*α*_3_·*α*_4_)^{1/4}^ = 0.72 (example values).

Step 6: *R*_carrier = (a·b·c·d)^{1/4}^ = 0.68 (example values).

Step 7: *H* = *R*_carrier × *R*_consciousness = 0.72 × 0.68 = 0.49.

Step 8: *H*_trend = [*α*_1_]^2^ = (0.72)^2^ = 0.52.

Interpretation: *H* = 0.49 indicates moderate life fluency; *H*_trend > *H* suggests high growth potential.

The HCF framework has been implemented as a digital tool (PsychHealth Calculator). A pre-registered validation study is currently in progress. Preliminary data are consistent with the framework’s predictions, suggesting that the derived H metric is associated with physical and mental health outcomes. Full validation results will be reported separately.

## 5 Discussion

The preceding sections introduced the Holographic Coding Framework (HCF) as a theory-driven alternative to inductive scale development. The framework meets the four necessary conditions for theory-driven measurement: it begins with explicit axioms, defines its core constructs independently of any instrument, specifies a formal causal structure (*α*_1_ → *α*_2_ → *α*_3_ → *α*_4_), and provides a traceable mapping from observable behaviors to latent states via energy codes.

The HCF framework not only measures but also identifies intervention targets. Each *α*-factor points to a distinct intervention direction: low *α*_1_ (metacognitive clarity) calls for practices that enhance self-observation; low *α*_2_ (emotional acceptance) calls for acceptance-based training; low *α*_3_ (self-consistency) calls for conflict resolution and cognitive restructuring; low *α*_4_ (goal-directed efficacy) calls for action-focused support. Thus, unlike traditional scales that provide a score without a clear intervention pathway, the HCF framework directly links measurement to actionable targets.

To clarify how HCF differs from conventional measurement paradigms, Table 5 contrasts its core features with traditional scale-based approaches.

**Table 5:** Comparison of measurement paradigms: traditional scale-based vs. HCF.

Dimension	Traditional scale-based measurement	HCF (theory-driven)
Starting point	Item pool + factor analysis	Axiomatic assumptions (*K* ≡ 1, dual-layer, embodiment, asymmetry)
Construct definition	Defined by the scale (circular)	Defined independently (*α*_1_–*α*_4_, a–d carriers)
Relation to language	Inseparable from item wording	One behavioral indicator among many
Semantic confound	Cannot be avoided	Bypassed via indirect mapping (energy codes → *α*-factors)
Testable predictions	Factor structure, reliability, validity correlations	*H*_trend = [*α*_1_]^2^, *α*_2_ threshold, *α*_3_ cutoff
Growth prediction	No	Yes: *H*_trend = [*α*_1_]^2^

The comparison highlights several distinctive features of the HCF approach. First, unlike traditional scale development which begins with item pools and factor analysis, HCF starts from axiomatic assumptions about the nature of consciousness-carrier systems. Second, HCF defines its constructs (*α*_1_–*α*_4_, a–d carriers) independently of any measurement instrument, breaking the circularity of construct definition that plagues inductive scale development. Third, by mapping behavioral responses to energy codes and then to α-factors, HCF bypasses the semantic confound that affects self-report measures. Fourth, HCF generates testable predictions that go beyond factor structure and reliability, including a quadratic growth prediction (*H*_trend = [*α*_1_]^2^) and threshold effects (e.g., *α*_2_ > −0.5). These predictions have been pre-registered (OSF d4e7p) and are currently being tested in an ongoing validation study using the PsychHealth Calculator.

The HCF is not proposed as a replacement for all existing measures but as an illustration of what theory-driven measurement can look like. Its principles may be instantiated in other frameworks as well. The framework remains under development, and its empirical validation is ongoing. However, even at this stage, the HCF demonstrates that it is possible to design a measurement system that begins with theory, defines constructs independently of items, and generates falsifiable predictions—thereby avoiding the semantic confound that undermines traditional scale validation.

## 6 Implications and limitations

### 6.1 For scale developers

The most immediate implication of our analysis is that scale development should begin with theory, not with items. Before writing a single item, researchers should specify what the target construct is, how it manifests in observable behavior, and what causal role it plays in the broader psychological system. Items should be generated to test whether theoretically predicted behavioral patterns exist, not to maximize internal consistency.

### 6.2 For reviewers and editors

Journals that publish scale development and validation studies should require authors to articulate the theoretical basis of their constructs, not merely report psychometric indices. A manuscript that reports excellent fit statistics for a scale whose theoretical foundations are unclear should be treated with the same skepticism as a manuscript with poor fit statistics.

### 6.3 For journal policy

The SOBER guidelines (Simons et al., 2017) called for standardized measurement practices to address the proliferation of idiosyncratic scales. Our analysis suggests that standardization alone is insufficient. Standardizing poor measurement—measurement that captures semantic associations rather than psychological states—does not solve the validity crisis; it entrenches it. Theorization must precede standardization.

### 6.4 Limitations

This paper has argued that the root cause of the measurement crisis is theoretical, not statistical. We have not provided a complete theory that resolves this crisis—such a theory would require a scope far beyond the present analysis. We have also not empirically demonstrated that theory-driven measurement outperforms traditional methods; such a demonstration would require a fully validated theory-driven measure, which is currently under development and will be the subject of future research.

These limitations are substantial. But they also define a research agenda. The first step toward solving the measurement crisis is recognizing that it exists and understanding its causes. We hope this conceptual analysis contributes to that recognition.

A pre-registered validation study using the PsychHealth Calculator is currently in progress to address these limitations.

## Data Availability

The analysis code and pre-registrations for the empirical studies cited in support of the theoretical framework will be made publicly available on the Open Science Framework upon publication.
